# Predicting the growth situation of *Pseudomonas aeruginosa* on agar plates and meat stuffs using gas sensors

**DOI:** 10.1038/srep38721

**Published:** 2016-12-12

**Authors:** Xinzhe Gu, Ye Sun, Kang Tu, Qingli Dong, Leiqing Pan

**Affiliations:** 1 College of Food Science and Technology, Nanjing Agricultural University, No.1, Weigang Road, Nanjing, Jiangsu 210095, PR China; 2 School of Medical Instrument and Food Engineering, University of Shanghai for Science and Technology, 516 Jun Gong Rd., Shanghai 200093, PR China

## Abstract

A rapid method of predicting the growing situation of *Pseudomonas aeruginosa* is presented. Gas sensors were used to acquire volatile compounds generated by *P. aeruginosa* on agar plates and meat stuffs. Then, optimal sensors were selected to simulate *P. aeruginosa* growth using modified Logistic and Gompertz equations by odor changes. The results showed that the responses of S_8_ or S_10_ yielded high coefficients of determination (R^2^) of 0.89–0.99 and low root mean square errors (RMSE) of 0.06–0.17 for *P. aeruginosa* growth, fitting the models on the agar plate. The responses of S_9_, S_4_ and the first principal component of 10 sensors fit well with the growth of *P. aeruginosa* inoculated in meat stored at 4 °C and 20 °C, with R^2^ of 0.73–0.96 and RMSE of 0.25–1.38. The correlation coefficients between the fitting models, as measured by electronic nose responses, and the colony counts of *P. aeruginosa* were high, ranging from 0.882 to 0.996 for both plate and meat samples. Also, gas chromatography–mass spectrometry results indicated the presence of specific volatiles of *P. aeruginosa* on agar plates. This work demonstrated an acceptable feasibility of using gas sensors—a rapid, easy and nondestructive method for predicting *P. aeruginosa* growth.

A vital food microorganism, *Pseudomonas aeruginosa* is a common opportunistic human pathogen that is widely distributed in the environment; it plays an important role in the spoilage of a variety of foods^1^,^2^. There is an urgent need to develop a rapid, accurate and sensitive method to predict the quality of *P. aeruginosa* in foods, especially in high value foods such as meat. Generally speaking, aerobic storage of meat allows *Pseudomonas* spp. to become a dominant spoilage bacterium at different storage temperatures^3^. It was reported that *Pseudomonas* spp. were specific spoilage organisms (SSO) in chilled meat, which had an obvious advantage in the growth rate over other bacteria under aerobic conditions^4^,^5^. SSO referred to the microbe that mainly caused food sources spoilage and made them produce off flavors in the process of spoilage^6^. It’s feasible that SSO was chosen to establish mathematical models used for predicting the decay of food because only few species and strains, namely SSO, in the initial microbial association caused the spoilage of food sources. Also, Egan^7^ pointed out the spoilage in chilled meat was mainly caused by aerobic gram-negative bacteria under aerobic conditions, which was simply regarded as caused by *Pseudomonas* spp. to a certain extent.

Currently, predictive microbiology is a powerful tool in predicting microbiological changes in food, which aims to establish mathematical models used for describing dynamic changes of microorganism over time under specific conditions^8^. Many factors have an important influence on the growth situation of microorganism, including factors that arise during the processing and distribution of the food (i.e., temperature, humidity, packing conditions, etc.) or that pertain to the natural characteristics of the food (pH value, sensory score, etc.)^9^. Studies have been done that produced kinetic models for predicting the growth situation of microorganism, especially lag time and maximum specific growth rate. In recent years research has focused on predicting spoilage microorganisms in food in this field^10^. Certain mathematical models are reported to provide a qualitative or quantitative description for dynamic changes of *Pseudomonas* spp^10^. For, example, Walter^13^ established kinetic models for *Pseudomonas fluorescens* under various treatment conditions in whole milk using a modified Logistic equation. Li *et al*.^14^ evaluated fitting growth models of *Pseudomonas* spp. at different temperatures using both modified Gompertz and Huang equations. However, these studies, which are based on microbiological analysis, suffer from several defects (i.e., a large amount of pretreatment work, the large number of elements required for the operation, a long period of training, the delay in obtaining results, etc.), especially when conventional microorganism counting is involved. Thus, we must develop a method for monitoring and predicting the growing situation of *Pseudomonas* spp. in food that is easier and faster than the conventional microbiological analysis method.

Odor is a sensitive index in the food industry, and is especially important when decay or decomposition occurs in foodstuffs due to microbial infection. Gas sensors, usually called an “electronic nose,” have been widely used for detecting and determining the quality of microorganisms in food^15^,^16^,^17^. As is generally known, microorganisms produce many different kinds of volatile compounds (microbial volatile organic compounds, or MVOCs)^18^. MVOCs are important components of microbiological metabolites and contain abundant biological information^19^. Hu *et al*.^18^ evaluated microbial volatile metabolites of three *Pseudomonas* species and provided an important differentiation of these three strains using an electronic nose combined with GC-MS. A more specific profile regarding MVOCs may be supplied using GC-MS, but that method consumes a great deal of time and labor. Gas sensors are a more practical tool for monitoring microorganisms due to their sensitivity to certain specific MVOCs. Previous studies have applied gas sensors to detect MVOCs and identify the species of food microorganism that produced them. Gutiérrez-Méndez^20^ evaluated the aroma generation of *Lactococcus lactis* strains using an electronic nose and successfully classified 23 strains into 4 distinctive groups according to their aroma profiles. McEntegart^21^ similarly discriminated two strains of *Escherichia coli* using a special array of gas sensors. This discrimination also exists in volatiles that are distributed by food infected with different strains of microorganisms, and this confirms the feasibility of using gas sensors to detect and determine the quality of different strains of microorganisms in food. Lippolis^22^ *et al*. classified different *Penicillium* strains according to whether they produced ochratoxin A using a MOS-based electronic nose, and he achieved recognition percentages of greater than 82%. Also, there was qualitative or quantitative relationship between the odor and colony counts in food. Wang^23^ *et al*. established prediction models for different *Zygosaccharomyces rouxii* strains in apple juice using an electronic nose, with a correlation coefficient in the range of 0.97–0.98. However, almost all of these studies were aimed solely at successfully classifying the different species, quantity, and metabolites of microorganisms using an electronic nose, or at establishing the relationship between the properties of the microorganisms and their smell fingerprint. No studies have been done to date that show that the dynamic growth situation of microorganisms may be predicted by odor fingerprint information. Some kinetic parameters in particular play an important role in microorganism changes over time, such as lag time and maximum specific growth rate. They are vital for food safety but difficult to describe and predict based on conventional microbiological analysis in predictive microbiology^24^. A large amount of research has been focused on developing kinetic models and parameters of *Pseudomonas* spp. in meat using microbiological analyses, but these models are time-consuming and laborious methods of modeling *Pseudomonas* spp. growth^25^.

Therefore, in our work, gas sensors were used to establish kinetic models of *P. aeruginosa* for predicting the growth situation. Simultaneously, odor profiles provided by *P. aeruginosa* inoculated on an agar plate were analyzed by headspace solid phase micro-extraction (HS-SPME) and GC-MS, were used to seek characteristic volatiles of this strain. The purpose of this work was to select optimal sensors to simulate the growth kinetics of *P. aeruginosa* on an agar plate and in meat stuff, and then to evaluate these dynamical models and kinetic parameters based on selected sensors in comparison with models based on colony counts of *P. aeruginosa*.

## Results

### Growth simulation of *P. aeruginosa* on agar plates by gas sensors

#### Gas sensors response

**Figure 1:**
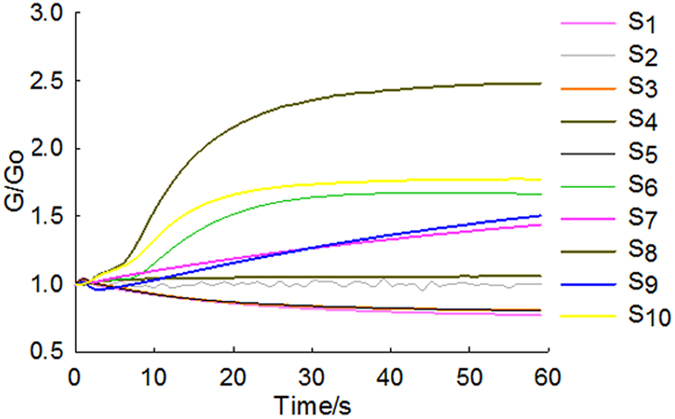
Response values of 10 sensors of an agar plate sample inoculated with *P. aeruginosa* at 36 h.

Figure 1 shows the response values of 10 gas sensors of an agar plate sample inoculated with *P. aeruginosa* for 36 h. Each response curve represents the G/G_0_ value of a single sensor when a sample gas entered in the chamber. G_0_ and G respectively represented the conductance of the 10 sensors in touch with clean air and the sample gas in the headspace. During the course of the measurement, the G/G_0_ value of most sensors gradually deviated from the initial value (G/Go = 1) in most of the samples. At the beginning of the measurement (0–20 s), the conductivity of each sensor constantly changed; it was gentle at 30 s and reached a peak at 55 s, apart from S_2_ and S_4_. Responses of 10 sensors of the control group (CK) had smaller changes over time and they were not regular (Supplementary Fig. A1). Because all sensors showed a stable response value in the time period from 55 s to 58 s, the response value at 58 s was chosen for the data analysis. As Fig. 1 shows, S_8_, S_10_ and S_6_ presented higher response values and experienced more significant changes than the other sensors, suggesting that these three sensors were more sensitive to the volatile compounds of *P. aeruginosa* on the agar plates.

#### Sensor selection

Selection of the sensors applied to the growth simulation of *P. aeruginosa* depended mainly on whether they responded significantly to the volatile compounds of the plates inoculated with *P. aeruginosa*. The contribution of 10 sensors combined with the volatile compounds of plates inoculated with *P. aeruginosa* was weighed by loading analysis. The results showed that the first principal component of loading analysis (LA 1) accounted for 84.19% variance and determined each of the 10 sensors on the contribution. Thus, the combination of the sensors with the highest response included S_8_, S_6_, S_10_, S_9_ and S_7_ according to LA 1 (Supplementary Fig. A2).

Duncan’s multiple comparison test (*P* < 0.05) of one-way ANOVA by SPSS 18 (SPSS, Inc., Chicago, IL, USA) was carried out to evaluate whether the performance of the 10 sensors differed significantly in detecting the volatiles generated by *P. aeruginosa* at different detection points during 48 h of incubation on the plate (Supplementary Table B1). If one sensor achieved better discrimination over the entire course of the incubation process, then it should be selected for use in the growth simulation of *P. aeruginosa* on the plate^26^. The results demonstrated that only S_5_ met the full differentiation criteria across all of the detecting points in the incubation. S_1_, S_3_, S_7_, S_8_ and S_9_ each distinguished four detecting points during the whole incubation. S_6_ and S_10_ produced significantly different results at the three detecting points. S_2_ and S_4_ found only a small difference in the volatile compounds generated by *P. aeruginosa* at different detecting points.

Pearson correlation analysis was performed to measure the linear relationship between the signals of the 10 sensors and the colony counts of *P. aeruginosa* during the incubation on the plate (Supplementary Table B2). According to the results, S_8_ and S_10_ formed a comparatively highly correlated block, yielding (respectively) a logarithmic (base 10) function of Colony Forming Unit (CFU) of *P. aeruginosa* of 0.885 and 0.942. However, other sensors displayed a low correlation with the logarithm of CFU in a range of 0.151–0.525. Although several sensors (i.e., S_6_ and S_5_) produced a more significant contribution or found significant differences in the volatiles generated by *P. aeruginosa* during the incubation, the change trends of the response value of these sensors were inconsistent with the actual growth situation of the bacterium. Hence, they were not taken into consideration. Considering the results of the load analysis, variance analysis and Pearson correlation analysis, only S_8_ and S_10_ were preliminarily identified as sensors suitable for growth simulation prediction of *P. aeruginosa* on the agar plate.

#### Growth simulation of P. aeruginosa on agar plates

**Figure 2:**
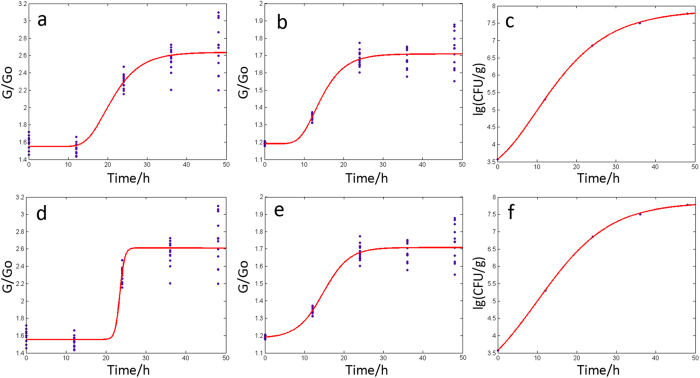
Fitting the growth curve for *P. aeruginosa* on the agar plate (**a**) S_8_, Gompertz; (**b**) S_10_, Gompertz; (**c**) lg(CFU/g), Gompertz; (**d**) S_8_, Logistic; (**e**) S_10_, Logistic; (**f**) lg(CFU/g), Logistic).

According to the results of the sensor selection, S_8_ and S_10_ were employed to simulate the growth situation of *P. aeruginosa* on the agar plate using modified Gompertz and Logistic models. The two mathematical models were the most typical and basic models for simulating microbial growth^27^,^28^,^29^. The simulation equations and model parameters of S_8_ and S_10_ are displayed in Table 1; the growth curves of S_8_ and S_10_ using Gompertz and Logistic models are shown in Fig. 2(a–f). The fitting growth models from two sensors produced high R_c_^2^s, in a range of 0.89–0.93, and a low RMSE_C_, with the values ranging between 0.06–0.17 based on modified Gompertz and Logistic models. Therefore, the model for the training data set of S_10_ had better simulation accuracy, with higher Rc^2^ values of more than 0.9 and a lower RMSE_C_ of 0.06. Following validation by the testing dataset, the model parameters of S_10_ were 0.9499 (R_p_^2^, Gompertz), 0.9908 (R_p_^2^, Logistic), 0.0505 (RMSE_P_, Gompertz) and 0.0499 (RMSE_P_, Gompertz), respectively, which were superior to the R_p_^2^s and RMSE_P_ of S_8_ in the test dataset using the two modified models.

The colony count of *P. aeruginosa* on the agar plates was calculated according to China’s national standard (GB) method called “Food microbiological examination: Enumeration of coliforms”^30^. In this study, the logarithmic (base 10) of CFU of *P. aeruginosa* was employed to simulate the Gompertz and Logistic models. The simulation equations of lg(CFU/g) are displayed in Table 1. Further, the two simulation models both had very high R^2^ values of up to 0.999, providing an accurate simulation for the growth stages of *P. aeruginosa*. In addition, the fitting growth models of S_8_ and S_10_ were compared to other models in terms of the logarithmic of the CFU of *P. aeruginosa* using the correlation coefficients (r) with values of 0.9377 (S_8_, Gompertz), 0.9346 (S_8_, Logistic), 0.9759 (S_10_, Gompertz) and 0.9754 (S_10_, Logistic), respectively. The response of S_10_ supplied a relatively better fitting growth model for the growth stages of *P. aeruginosa* on the agar plates. Some growth kinetic parameters are listed in Table 1, including lag time (λ) and maximum specific growth rate (μ_max_). Pronounced lag time was evident for the fitting growth models performed by individual sensors. However, there was no lag time for the curve simulation model of the logarithmic of the CFU using the modified Gompertz model. That model mainly referred to a scattered measurement of culture time and appropriate culture conditions, which caused the model to miss the detection of a lag phase of the *P. aeruginosa*. As Table 1 shows, the lag phase of the growth models produced by the sensors was longer than the models of the colony count of *P. aeruginosa* produced by two mathematic models. In particular, S_8_ indicated that changes in the odor fingerprint were generated by the growth and activities of *P. aeruginosa*. Comparatively speaking, the dynamic variation of S_10_ was closer to the logarithmic of the CFU than that of S_8_. Hence, S_10_ supplied a more accurate simulation model for the growth of *P. aeruginosa* on the agar plate.

#### Classification of agar plate samples by principal component analysis

**Figure 3:**
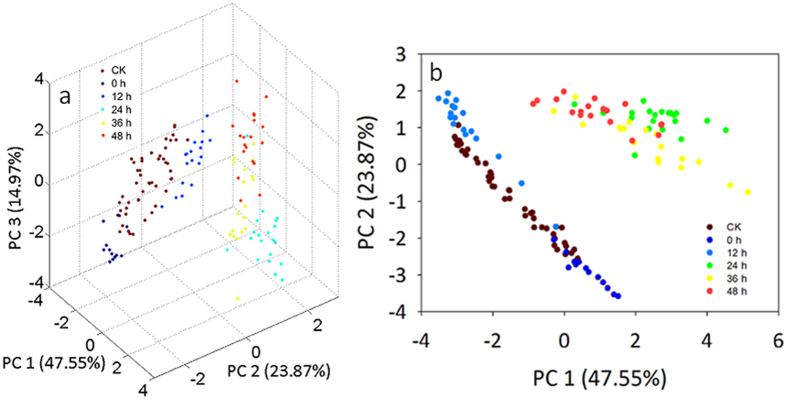
The PCA discriminating results of agar plate samples inoculated with *P. aeruginosa* at different detecting points in the incubation of 48 h (**a**: 3D plot; **b**: 2D plot).

Principal component analysis (PCA) translates multiple variables that have strong pertinence into several comprehensive variables that are unrelated to each other using linear combination^31^. Generally, if the accumulative contribution of certain PCs reached 85%, these PCs can stand in for most of the information of the original data. PCA was employed to classify the growth situation of *P. aeruginosa* on the agar plate at different points in time (i.e., 0 h, 12 h, 24 h, 36 h and 48 h) using the signals of the 10 sensors. As seen in the PCA 3D plot (Fig. 3a), the first three PCs accounted for 47.55%, 23.87% and 14.97% of the total variance, respectively. The control group (CK) contained 50 samples during the entire incubation period of 48 h (10 samples for each detecting point). The data points of the agar plate samples at 0 h, 12 h and 24–48 h were gathered, and appeared to have a clear separation from each other in the PCA 2D plot (Fig. 3b). Also, it could be seen that data points of the plate samples at 24 h were separated from those at 36 h and 48 h in Fig. 3a, except for certain individual data points. This reflects the difference in the volatile compounds generated by *P. aeruginosa* on the agar plate at the four periods (0 h, 12 h, 24 h and 36–48 h). The data points of the plate samples at 36 h and 48 h partially overlapped, suggesting that there was a little difference in the volatile compounds generated by *P. aeruginosa* on the agar plate at 36 and 48 h. Finally, except for some overlap with data points at 0 h and 12 h, the control groups appeared to have a clear separation from the plate samples inoculated with *P. aeruginosa*.

The PCA results were in accordance with the growth tendency of *P. aeruginosa* on the agar plate. During 48 h of incubation, the colony counts of *P. aeruginosa* experienced lag, logarithmic and stationary phases in accordance with the consumption of the nutrients of the medium. When the bacterium had grown continuously to a certain extent, it began to produce water-soluble green metabolites and characteristic red pigments^32^. At a specific period, the colony counts of the *P. aeruginosa* reached a particular number, and specific metabolites were produced that sent out specific volatiles^33^. Thus, the composition of volatiles generated by the agar plates inoculated with *P. aeruginosa* was related to the consumption of the bacterium in the culture medium and the production and accumulation of the metabolite. This indicates that there was an apparent difference in the volatile compounds generated by *P. aeruginosa* on the agar plate at most of the later detection points.

#### HS-SPME/GC–MS analysis

The volatile compounds of the agar plate samples inoculated with *P. aeruginosa* were analyzed by HS-SPME/GC–MS at 0, 12, 24, 36 and 48 h of incubation, respectively. As shown in Table 2, 16 volatile compounds were identified on the agar plate samples inoculated with *P. aeruginosa*. They belonged to different chemical classes, including sulphur compounds, aromatic compounds, aldehydes, olefin, alkane, alcohols, esters and ethers (Table 2). According to existing research, the volatile compounds of microorganisms possess species specificity, which may serve as marker compounds to identify the species^34^.

For these reasons, disulfide, dimethyl, pyrazine, 2,5-dimethyl-, cyclopropane, 1-methyl-2-octyl and cetene were positively correlated with the presence of *P. aeruginosa*, because these compounds appeared in later periods of incubation (after 24 h) (Supplementary Fig. A3), suggesting that they may permit differentiation of the agar plate samples in terms of whether they were inoculated with *P. aeruginosa*. Hu *et al*. detected disulfide, dimethyl and pyrazine, 2,5-dimethyl- in tryptic soy broth (TSB) inoculated with *P. aeruginosa* after 18 h of incubation^18^. Wang discovered that a specific volatile compound in meat samples inoculated with *Pseudomonas* spp. was methy1 sulfide^35^, but it was not detected in this study. In particular, benzaldehyde, nonanal, hexadecanal, 2-tetradecanone, decanal, and dodecanal only appeared at 0 h during the incubation. Benzaldehyde, nonanal and decanal were detected in the control group during the 48 h incubation (Supplementary Table B3). Hence, it may have been the case that these compounds were diffused by the culture medium, not generated by the consumption of the bacterium inoculated with *P. aeruginosa* or the production and accumulation of the metabolite. Certain volatile compounds, such as 1-Hexanol and 2-ethyl-, appeared the early and later periods of incubation, but disappeared in the interim period. It was confirmed by Wang^35^ that this phenomenon for 1-hexanol and 2-ethyl- similarly existed in meat samples inoculated with *Pseudomonas* spp.

The HS-SPME/GC–MS results indicated that there was a great difference in the composition of volatile compounds generated by agar plate samples inoculated with *P. aeruginosa* at different detection points. This confirmed the feasibility of using an electronic nose to differentiate different growth stages of *P. aeruginosa* on the agar plate.

### Growth simulation of *P. aeruginosa* inoculated in fresh pork by gas sensors

#### Gas sensors response changes to P. aeruginosa in pork stored at 20 °C

**Figure 4:**
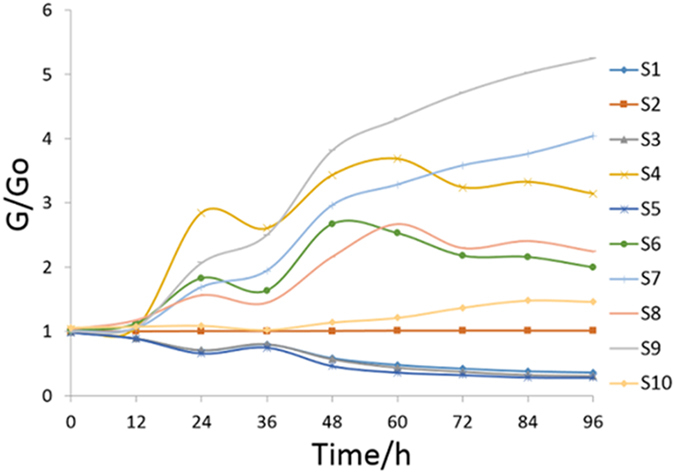
Average response values of 10 sensors of meat samples inoculated with *P. aeruginosa* at 20 °C during the storage of 96 h.

An odor fingerprint map of *P. aeruginosa* in pork stored at 20 °C from 0 h to 96 h generated by gas sensors is exemplified in Fig. 4. Each curve represents the average response values of 20 replicate measurements for a single sensor in a 10-sensor array. Initially, the G/G_0_ values of the 10 sensors changed slightly (increased or decreased) in the first few hours (0–12 h). Then, the response values of all sensors appeared to have a sharp change after 12 h, especially S_4_ and S_9_. However, there was a significant reduction in certain volatile compounds to which several sensors (S_4_, S_6_ and S_8_) were sensitive (24–36 h). During the following storage period, the responses of most sensors increasingly deviated from the initial G/Go radio; however, the responses of S_4_, S_6_ and S_8_ did not. At 96 h, S_9_, S_7_ and S_4_ presented the highest response values. During the entire storage period of 96 h, three sensors (S_9_, S_7_ and S_4_) showed more significant changes than the other sensors, suggesting that they were more sensitive to the volatile compounds generated by *P. aeruginosa* in pork.

#### Sensor selection for growth simulation of P. aeruginosa in fresh pork stored at 20 °C

In the same way, sensors were selected by loading analysis, variance analysis and Pearson correlation analysis for the growth simulation of *P. aeruginosa* in fresh pork stored at 20 °C. The result of the loading analysis demonstrated that LA 1 and LA 2 accounted for 90.38% and 7.32% variance. According to the rule that the selected sensors should have a higher contribution, the particular combination used was S_9_, S_7_, S_4_ and S_5_ for growth simulation (Supplementary Fig. A4).

Significance analysis of the signals of 10 sensors for fresh pork stored at 20 °C inoculated with *P. aeruginosa* was performed using one-way ANOVA (Duncan, *P* < 0.05) (Supplementary Table B4). Statistical analyses revealed that the response values of all sensors were significantly different at different detecting points. As described above, the selection of the optimal sensors should follow the rule that the sensors achieve the largest amount of differentiation during the entire storage period of 96 h as quickly as possible. The results showed that only S_9_ met the full differentiation among all of the detecting points during the 96 h period. S_1_, S_3_, S_5_, S_6_, S_7_ and S_8_ all differed significantly between each pair of 8 detecting points (9 points in all). Thus, the combination of S_1_, S_3_, S_5_, S_6_, S_7_, S_8_ and S_9_ showed the best discrimination performance over the entire course of the 96 h storage period.

The results of Pearson correlation analysis demonstrated that there was a highly correlated block between the signals of certain sensors and the colony counts of *P. aeruginosa* in the pork during the 96 h storage period (Supplementary Table B5). For example, S_1_, S_3_ and S_5_ appeared to have a high negative correlation (greater than 0.94); meanwhile, S_7_, S_8_ and S_9_ appeared to have a high positive correlation, in a range from 0.916–0.962. And S_4_ provided a relatively higher positive correlation greater than 0.85. Generally speaking, the change trends of the response values of the six sensors (S_1_, S_3_, S_4_, S_5_, S_7_, S_8_ and S_9_) were in accordance with the growth situation of the bacterium in the pork. These seven sensors should be taken into consideration as the optimal sensors for the growth simulation of *P. aeruginosa* in fresh pork. However, there is a regulation that the Pearson correlation between sensors could not exceed 0.99 because of overlaps in response values. The values among the three sensors (S_1_, S_3_ and S_5_) all exceeded 0.99. The other combination (S_7_ and S_9_) had a positive correlation with a value of 0.994. Therefore, only one sensor in each of the two groups was selected to stand for its group’s combined results. Considering the results of the load analysis and variance analysis, sensors S_5_ and S_9_ had a higher contribution and showed a more significant difference in response signals. Finally, the combination of S_9_, S_5_, S_4_ and S_8_ was chosen for the growth simulation of *P. aeruginosa* in fresh pork stored at 20 °C.

#### Growth simulation of P. aeruginosa in fresh pork stored at 20 °C

In our work, sensors S_9_, S_5_, S_8_ and S_4_ were determined to have the best performance in simulating *P. aeruginosa* growth in fresh pork stored at 20 °C based on loading analysis, variance analysis and Pearson correlation analysis (Supplementary Fig. A5). In the same way, the modified Gompertz and Logistic models were applied to the fitting growth situation of *P. aeruginosa*. Since the response values of S_5_ showed a negative correlation with the colony count, its negative values were employed to establish the fitting growth models. Based on S_9_ and S_5_, the training data set had a good fit with *P. aeruginosa* growth in fresh pork with a high Rc^2^ (greater than 0.92) and a low RMSE_C_ (in a range of 0.06–0.36) using two mathematical models in Table 3. These four models were validated by the testing data set, indicating a slightly decreased Rp^2^ for S_5_ (with a value of approximately 0.89) using both Gompertz and Logistic models. Also, the responses of S_8_ and S_4_ provided some fitting growth models of *P. aeruginosa* in fresh pork with an R^2^ of 0.81–0.87 using both training and testing data sets. In addition, because the first principal component (PC 1) of the response values of 10 sensors accounted for 80.4% of the total variance, the scores of PC 1 were chosen to simulate the growth of *P. aeruginosa* in fresh pork. The results (Table 3) indicated that PC 1 fit well with the growth of *P. aeruginosa* using two mathematical models. The two models produced by the training data set of PC 1 both had a higher Rc^2^, with a value of approximately 0.93. After being evaluated against the testing data set, PC 1 also provided an accurate simulation with an Rp^2^ of approximately 0.91.

The colony counts of *P. aeruginosa* in fresh pork were determined by a basic method described by Olga S. Papadopoulou^3^. The simulation equations of the logarithmic (base 10) of CFU are displayed in Table 3. The data points of lg(CFU/g) presented a very satisfactory simulation of *P. aeruginosa* growth, with an R^2^ greater than 0.99 for both the Gompertz and Logistic models. The fitting growth models produced by four sensors and PC 1 were compared with models using the logarithmic of CFU of *P. aeruginosa*. S_9_, S_5_ and PC 1 all supplied a relatively better simulation of *P. aeruginosa* growth, with very high correlation of greater than 0.98. Some growth kinetic parameters of *P. aeruginosa* in fresh pork are listed in Table 3. Comparatively speaking, PC 1 supplied the most accurate simulation model for the growth of *P. aeruginosa* in fresh pork, because it had the closest lag time and maximum specific growth rate to lg(CFU/g). This was mainly because PC 1 accounted for 80.4% variance, colligating most of the information of the original signals from all of the sensors. Therefore, the PC 1 of the signals of 10 sensors was superior to that of the individual sensors for the growth simulation of *P. aeruginosa* in fresh pork.

#### Growth simulation of P. aeruginosa in fresh pork stored at 4 °C

Meat samples stored at 20 °C went bad rapidly and exhibited the apparent presence of off-flavors after 2 days. Hence, meat samples stored at 4 °C after inoculation were employed to study the growth situation of *P. aeruginosa* during a 240 h period of storage using gas sensors. The odor fingerprint of these samples experienced a similar change to those stored at 20 °C, but it was much slower. Likewise, sensors were selected according to the same processing methods (loading analysis, variance analysis and Pearson correlation analysis). Sensors S_8_, S_4_, S_9_, S_6_ and S_7_ had much more significant responses than the other sensors (Supplementary Fig. A6). Loading analysis shows that LA 1 accounted for 80.37% variance, and S_8_, S_4_, S_9_, S_7_ and S_6_ contributed more than the other sensors to the variance of LA 1 (Supplementary Fig. A7). All sensors, apart from S_2_, distinguished more than 5 detecting points out of the 11 detecting points during the entire 240 h storage period (Supplementary Table B6). Pearson correlation analysis displayed the correlation between the response values of the 10 sensors and the colony counts of *P. aeruginosa* (Supplementary Table B7). S_9_, S_4_ and S_7_ provided a relatively higher positive correlation, with values in the range of 0.848–0.896. At the same time, S_1_, S_3_ and S_5_ appeared to have a negative correlation of greater than 0.74, but these three sensors made the smallest contribution in terms of the variance of LA 1 and LA 2 as determined by loading analysis. In addition, although S_8_ had the largest response and made the highest contribution of all of the sensors, it provided a low correlation (0.578) with the colony counts of *P. aeruginosa*. Moreover, S_7_ and S_9_ had a very high correlation, with a value of 0.993. Hence, S_4_ and S_9_ were selected to simulate the growth of *P. aeruginosa* in fresh pork stored at 4 °C.

The modified Gompertz and Logistic models were applied to fitting the *P. aeruginosa* growth. Results are shown in Table 4, including the fitting models based on the colony counts and the response values of S_4_, S_9_ and PC 1 (Supplementary Fig. A8). Sensor S_9_ performed better in terms of fitting the *P. aeruginosa* growth, with an R_c_^2^ of 0.8322 and 0.8450, as measured by modified Gompertz and Logistic models, respectively. S_4_ and PC 1 provided a relatively worse simulation for the growth of *P. aeruginosa*, with R_c_^2^ in a range of 0.73–0.78 as measured by two mathematical models. However, these three fitting models (S_4_, S_9_ and PC 1) provided high correlations, with values in the range of 0.95–0.99, in comparison with colony count fitting models. Kinetic parameters, lag time and maximum specific growth rates are shown in Table 4. It can be seen that a single sensor (S_4_ or S_9_) provided a longer lag time than the colony count, but the PC 1 of the signals of the 10 sensors had a smaller value, as measured by two mathematical models.

## Discussion

Currently, various kinds of rapid diagnostic or screening technologies are employed to detect and identify food microorganisms; methods that are not destructive are used most often^8^. The use of gas sensors has been significantly developed through the interdisciplinary contributions of chemometrics, electricity, computers, etc. Here, gas sensors were employed to support the research in predictive microbiology. It aimed at establishing a series of mathematical models used for forecasting the growth of microorganism by changes of odor fingerprint, overcoming the shortcoming of the delay in obtaining results and a long period of training by traditional microbial detecting methods. Our work evaluated the odor profiles of *P. aeruginosa* on agar plates using a universal PEN3 electronic nose combined with GC-MS. Studies^3^,^36^,^37^ have established that *Pseudomonas* spp. is a common and typical dominant spoilage bacterium in food, including meat, vegetables and so on. In this study, signals of individual sensors were employed to simulate the growth situation of *P. aeruginosa* on an agar plate using modified Gompertz and Logistic models. Some kinetic parameters were provided based on odor models, such as lag time and maximum specific growth rate. As mentioned above, to date, there is no research that is similar to our work. In our study, S_10_ supplied the best fitting growth model of *P. aeruginosa* on an agar plate with an R^2^ greater than 0.9 and a RMSE of 0.04–0.07. When compared with models using lg(CFU/g), S_10_ reflected a significant ability to fit *P. aeruginosa* growth, with a high correlation of up to 0.97 by both modified Gompertz and Logistic models. Further, in this study, PCA results indicated *P. aeruginosa* on agar plates appeared a clear separation at most detection points during 48 h of incubation, which was mainly due to different MVOCs generated by the bacterium at different detection points over the course of the growth period. The work conducted by Gobbi proved that volatile compounds of microorganisms are quite different in variety and quantity^31^. HS-SPME/GC–MS results indicated that the specific volatile compounds were disulfide, dimethyl, pyrazine,2,5-dimethyl-, 1-Hexanol,2-ethyl-, cyclopropane,1-methyl-2-octyl and cetene. They appeared in the later stages of the growth period, and were considered to be characteristic components for distinguishing different growth stages of *P. aeruginosa* on an agar plate.

The growth situation of microorganisms in real food differs from that of the growth in a culture medium to some extent, because of the rich nutrients contained in real food. And the relationship between the odor and colony counts has always been a hot topic in the research regarding food spoilage, especially meat^38^. Many studies have explored their relationships and demonstrated that the colony counts could be predicted by the odor in food by some algorithms, such as support vector machine, neural network, partial least squares regression and so on^15^,^23^,^39^. These work confirmed there was high correlation between the two. In our work, pork, as an excellent example of high added value food, was used for the following measurements of simulating the growth situation of *P. aeruginosa*. *Pseudomonas* spp. is a dominant spoilage bacterium in meat during the aerobic storage^3^. The microbe can only survive on the surface of meat or at a depth of 3–4 mm under the surface due to its aerobiotic characteristics^40^. The growth of other bacteria in the chilled meat samples was inhibited because it could not compete with *Pseudomonas* spp. for effective oxygen molecules^41^,^42^. Hence, the corruption of chilled meat, including putrid odors, was usually caused by the activities of *Pseudomonas* spp. In this study, responses values of sensors were employed to established dynamical models of *P. aeruginosa* as conventional microorganism counting did with predicting food microorganism. Also, signals of several sensors appeared curve changes like “S” fitted both modified Gompertz and Logistic equations, which were the most typical and basic curves in predictive microbiology^13^,^25^. The simulating results showed that two sensors (S_9_, S_4_) and PC 1 fit well with the growth of *P. aeruginosa* in fresh pork as measured by two dynamic models (Gompertz and Logistic models), with an R^2^ of 0.73–0.96 and a RMSE of 0.25–1.38 for both the training and testing data sets. Compared with the actual microbial growth model using lg(CFU), all odor models provided higher correlation, in a range of 0.882–0.996. PC 1 in particular provided a high correlation (greater than 0.99) when combined with lg(CFU) in fitting the growth of *P. aeruginosa* in pork stored at 20 °C.

Currently, most research to date has only focused on establishing a relationship between microbial quantity and smell fingerprint. For example, Zaragozá^43^ built qualitative and quantitative models of mesophilic and psychrotrophic bacteria in Atlantic salmon using an optoelectronic nose. And Papadopoulou^3^ monitored the colony count of *Pseudomonas* spp., *Brochothrix thermosphacta* and *Enterobacteriaceae* in a beef filet during storage using a portable quartz microbalance-based electronic nose, building a strong correlation between microbial numbers and odor values. There have been no studies of the dynamics of microbial growth and describing the kinetic parameters based on odor models. Some kinetic parameters, such as the lag time and maximum specific growth rate, have great importance in monitoring microorganisms^24^. Especially the lag time was very difficult to be described in predicting the growth of microorganism in food. In this study, the lag time provided by PC 1 was relatively closer to the growth situation using lg(CFU). In the same way, PC 1 had the closest maximum specific growth rate to lg(CFU/g). Hence, the PC 1 of the 10 sensors may be employed to predict the dynamic growth situation of *P. aeruginosa* in pork, instead of using the traditional microbial count method. The results indicated that the use of gas sensors has the potential to be a promising microbiological monitoring technique in the future.

## Materials and Methods

### Bacterial strain

*P. aeruginosa* was provided by the College of Food Science and Technology at Nanjing Agricultural University of China. The strain was grown aerobically in a nutrient agar medium (10 g peptone, 3 g beef extract, 5 g sodium chloride, 1000 mL water, 20 g agar, pH 7.4 ± 0.1) at 37 °C and 85% RH for 3 days. Then, it was activated once again under the same culture conditions for 2 days. A single colony of a *P. aeruginosa* isolate was incubated in a 9 cm diameter agar plate. Then, a sterilized physiological saline solution (0.9% w/v NaCl) was used to gently wash the surface of the agar plate and a bacterial suspension of *P. aeruginosa* was obtained. In preparation for the following experiment, the concentration of the bacterial suspension was calculated with a hemocytometer using the formula (N/80 × 4 × 10^6^ × d), where N is the total number of bacterial cells in 80 small squares and d is the dilution factor of the bacterial suspension. The final concentration was adjusted to 10^4^ cells/mL.

### Sample preparation

150 agar plates were prepared by pouring 20 ± 1 g of the nutrient agar medium into the center of 9 cm petri dishes. After solidification and cooling, 100 plates were inoculated with 100 μL of bacterial suspension, while the remaining plates were inoculated with 100 μL of sterilized saline water as control group. Then, the plates were incubated at 37 °C and 85% RH for 48 h in an incubator at a constant temperature and humidity. Every 12 h, 30 plate samples (20 plates for *P. aeruginosa* and 10 for the control group) were analyzed using the electronic nose.

Fresh pork obtained from the longissimus dorsi muscles of different carcasses were purchased from the local meat market and transported to the laboratory within 15 min. The surface of the longissimus dorsi was firstly swabbed with 75% alcohol. And then it was divided into rectangular pieces (4 cm × 4 cm × 1 cm, 30 g for each) in a sterile environment, after removing the surface and taking out the internal meat on a clean bench. These rectangular pieces were soaked in a bacterial suspension of 10^4^ cells/mL of *P. aeruginosa* for 10 s, drained and then packed aerobically in 9 cm sterilized petri dishes with an air-permeable polyethylene plastic film. The petri dishes loaded with pork pieces were incubated at 20 °C and 50% RH for 96 h in an incubator at a constant temperature and humidity. There were a total of 180 meat samples and 20 samples for each detecting point (i.e., 0 h, 12 h, 24 h, ……, 96 h). 220 further meat samples were prepared using the same process and incubated at 4 °C and 50% RH for 240 h. Every 24 h, 20 samples were taken out for E-Nose measurement and microbiological examination.

### Electronic nose trials

The volatile compounds of the *P. aeruginosa* samples were determined using a portable electronic nose (PEN 3, Win Muster Air-sense Analytics Inc., Germany). The PEN3 E-nose consisted of a sampling system, a detection system and a pattern recognition system. The detection system was equipped with 10 metal oxide gas sensors that were partially selective toward different volatile compounds (Supplementary Table B8). Each sensor was generally sensitive to a specific set of volatile components (i.e., S_8_ was sensitive to alcohols and partially aromatic compounds). The pattern recognition system was used for data recording and analysis.

An agar plate inoculated with *P. aeruginosa* was placed in a 1000-mL beaker, sealed by tinfoil and conditioned at room temperature (20 °C ± 1 °C) until the volatile compounds in the headspace were equilibrated. In addition, a fresh pork piece inoculated with *P. aeruginosa* was placed in a 250-mL beaker and left at room temperature for 10 min to generate volatiles in the headspace. Following the cleaning process, the headspace gas from the beaker was injected into the sensor chamber at a constant rate of 300 mL/min. After the headspace gas entered the sensor chamber, the G/G_0_ ratio of the 10 metal oxide sensors immediately changed, which reflected the relative conductance of the sample gas in comparison with clean air. In the electronic nose trials, the total cycle time per sample was 3 min and 5 min for plate and meat samples, respectively. Volatile compounds from the agar plate samples were analysed by the electronic nose under the following conditions: cleaning time: 110 s; automatic zero setting time: 5 s; sample preparation time: 5 s; sample measurement time: 60 s; carrier gas: clean air (80% N_2_, 20% O_2_). For the meat samples, only the cleaning time was changed and set at 230 s in order to clean the remaining gas from the sensor chamber.

### HS-SPME/GC–MS trials

The volatile compounds of the agar plate samples were absorbed by HS-SPME, separated by GC (450 GC, Bruker, USA) and analyzed by MS (320-MS, Bruker, USA) at 0, 12, 24, 36 and 48 h of incubation. Following E-nose analysis, an agar plate was placed in a 250-mL beaker, sealed by tinfoil and conditioned at 30 °C in a water bath to enhance desorption of the volatiles from the agar plate into the headspace of the breaker. A PDMS/DVB fiber (poly-dimethylsiloxane, 65 μm, Supelco, USA) was aged in the GC injection port at 250 °C for 30 min and then exposed in the headspace of the agar plate sample for 60 min to extract volatile compounds. After extraction, the fiber was inserted into the GC injection port and thermally desorbed for 5 min. Volatile compounds were immediately transferred to the GC system and separated by a quartz capillary column (30 m × 0.25 mm i.d., 0.25 μm film thickness, BR-5ms, Bruker, USA). Initial column temperature was held at 50 °C for 3 min. Then, it was raised from 50 °C to 160 °C at a rate of 5 °C/min and maintained for 5 min. Next, it was increased to 250 °C at a rate of 13 °C/min and maintained for 2 min. The flow rate of the carrier gas (helium) was 1 mL/min. After separation, the volatile compounds were identified by the MS system. The ion source and the MS Quard were set at 230 °C and 150 °C, respectively. Electron impact mass spectra were recorded at 70 eV ionization energy. The mass scanning range was from m/z 30 to 450.

The volatile compounds of the agar plate samples were identified by a comparison of their mass spectra with data from the NIST library (2013) using a positive and negative match quality higher than 800. The relative content of every acquired compound was calculated by the percentage of the peak area that accounted for the total peak area of all acquired compounds. Each test set comprised three replicates and one control.

### Data analysis and mathematical modeling

In this study, odor fingerprint information was extracted using different data processing methods to predict the growth situation of *P. aeruginosa*. On the one hand, the signals of the individual sensors were extracted to simulate the growth of *P. aeruginosa*. On the other hand, the principal component scores of the response values of the 10 sensors were extracted by PCA (DPS14.5, Hangzhou Ruifeng information technology co., LTD, China) for simulating the growth of *P. aeruginosa*; this approach reduced superfluous information and simplified the calculation compared with the above processing method. PCA is a linear pattern recognition method and is widely used to reduce dimensionality and extracting features. In addition, the linear relationship between the odor fingerprint and the colony counts of *P. aeruginosa* was measured using Pearson correlation analysis (SPSS 18, Inc., Chicago, IL, USA). Loading analysis (Win Muster v.1.6 Air-sense Analytics Inc., Germany) was carried out to weigh the contribution of the 10 sensors. Duncan’s multiple comparison test (*P* < 0.05) of one-way ANOVA (SPSS 18, Inc., Chicago, IL, USA) was performed to determine the difference in the odor fingerprint of *P. aeruginosa* at different detection points.

In our work, two mathematical models were chosen to predict the growth situation of *P. aeruginosa* on agar plates and fresh pork samples using electronic nose data. These models were the modified Gompertz model (Eq. (1)) and Logistic model (Eq. (2)).


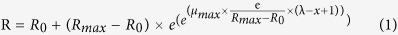



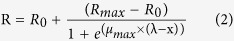


In Eq. (1) and Eq. (2), R (G/G_0_) was the response value of the sensor combined with sample gas; R_0_ and R_max_ (G/G_0_) were the initial response value and maximum response value, respectively; μ_max_ (h^−1^) was maximum specific growth rate of the response value; and λ (h) was the lag time of the response value.

Fitting the growth model of *P. aeruginosa* using electronic nose data was performed using the Curve Fitting Tool in MATLAB 2010b software (The Math Works Inc., Natick, USA). There were 20 data points at each detecting point for both agar plate and meat samples that were used to simulate the growth of *P. aeruginosa*. Among this data, 15 points were used to establish the mathematical model and the other 5 points were used for testing the model. The best fitting model was determined according to the following parameters: the coefficients of determination (R^2^) and root mean squared error (RMSE). Further, the performance of the fitting growth models using electronic nose data was evaluated by comparing the correlation coefficients (r) with the growth models based on colony forming units (CFU).

## Additional Information

**How to cite this article**: Gu, X. *et al*. Predicting the growth situation of *Pseudomonas aeruginosa* on agar plates and meat stuffs using gas sensors. *Sci. Rep.* **6**, 38721; doi: 10.1038/srep38721 (2016).

**Publisher's note:** Springer Nature remains neutral with regard to jurisdictional claims in published maps and institutional affiliations.

## Supplementary Material

Supplementary Information

## Figures and Tables

**Table 1:** Results of fitting growth models for *P. aeruginosa* on the agar plate.

Method	Sensor	Simulation equation	λ (h)	μ_max_(h^−1^)	Training			Testing			r
					R_c_^2^	RMSE_C_	SSE	R_p_^2^	RMSE_P_	SSE	
Gompertz	S_8_	f(x) = 1.55 + 1.087*exp(−exp(0.2246/1.087*(14.5-x) + 1))	14.5	0.0826	0.8963	0.165	1.497	0.9474	0.1192	0.426	0.9377
	S_10_	f(x) = 1.191 + 0.5178*exp(−exp(0.1283/0.5178*(8.79-x) + 1))	8.7	0.0472	0.9251	0.06067	0.2024	0.9499	0.0505	0.0764	0.9759
	lg(CFU/g)	f(x) = 3.207 + 4.671*exp(−exp(0.4527/4.671*(−0.6121-x) + 1))	—	0.4527	0.9999	0.02808	0.7*10^−4^	—	—	—	−
Logistic	S_8_	f(x) = 1.554 + 1.058/(1 + exp(1.694*(23.48-x)))	23.48	1.058	0.8937	0.1671	1.535	0.9454	0.1215	0.4425	0.9346
	S_10_	f(x) = 1.186 + 0.5225/(1 + exp(0.305*(14.75-x)))	14.75	0.5225	0.9249	0.06073	0.2028	0.9908	0.0499	0.0747	0.9754
	lg(CFU/g)	f(x) = 2.13 + 5.718/(1 + exp(0.1096*(9.955-x)))	9.955	5.718	0.9999	0.04079	1.664*10^−3^	—	—	—	—

**Table 2:** Volatile compounds (n = 16) identified by HS-SPME/GC–MS analysis of agar plate samples inoculated with *P. aeruginosa* during 48 h of incubation.

No	r_t_^a^	Volatile Compound	0 h	12 h	24 h	36 h	48 h
1	2.88	Disulfide,dimethyl	n.d.^b^	n.d.	n.d.	22.661 ± 5.328	22.836 ± 5.642
2	7.20	Oxime-,methoxy-phenyl-	n.d.	n.d.	n.d.	0.485 ± 0.429	n.d.
3	7.58	Pyrazine,2,5-dimethyl-	n.d.	n.d.	n.d.	0.555 ± 0.238	0.431 ± 0.380
4	9.63	Benzaldehyde	1.898 ± 0.303	n.d.	n.d.	n.d.	n.d.
5	11.61	1-Hexanol,2-ethyl-	n.d.	5.736 ± 0.505	n.d.	8.256 ± 2.014	10.407 ± 1.969
6	13.74	Cyclopropane,1-methyl-2-octyl	n.d.	n.d.	3.055 ± 3.234	11.341 ± 6.127	11.059 ± 4.620
7	14.14	Nonanal	3.112 ± 0.409	n.d.	n.d.	n.d.	n.d.
8	14.40	1-Decene	n.d.	n.d.	n.d.	1.095 ± 0.306	n.d.
9	16.19	Hexadecanal	0.447 ± 0.486	n.d.	n.d.	n.d.	n.d.
10	16.84	Dodecane	n.d.	0.133 ± 0.007	n.d.	0.546 ± 0.520	0.279 ± 0.070
11	16.86	2-Tetradecanone	0.272 ± 0.038	n.d.	n.d.	n.d.	n.d.
12	17.26	Decanal	4.093 ± 1.052	n.d.	n.d.	n.d.	n.d.
13	20.14	Dodecanal	0.723 ± 0.566	n.d.	n.d.	n.d.	n.d.
14	20.28	Oxirane, decy1-	n.d.	0.92 ± 0.007	n.d.	0.313 ± 0.136	1.042 ± 1.050
15	24.43	5,9-Undecadien-2-one,6,10-dimethyl- (E)-,	0.636 ± 0.398	0.279 ± 0.002	n.d.	0.241 ± 0.171	0.221 ± 0.004
16	25.89	Cetene	n.d.	n.d.	n.d.	n.d.	0.588 ± 0.617

^a^Rt: retention time.

^b^N.d.: not detected.

**Table 3:** Results of fitting growth models for *P. aeruginosa* in fresh pork stored at 20 °C.

Method	Sensor	Simulation equation	λ (h)	μ_max_(h^−1^)	Training			Testing			r
					Rc^2^	RMSE_C_	SSE	Rp^2^	RMSE_P_	SSE	
Gompertz	S_9_	f(x) = 0.9195 + 4.522*exp(−exp(0.2208/4.522*(13.34-x) + 1))	13.34	0.08124	0.9560	0.3485	15.79	0.9428	0.3824	6.580	0.9869
	S_5_	f(x) = −1.021 + 0.8115*exp(−exp(0.03248/0.8115*(1.662-x) + 1))	1.662	0.01185	0.9297	0.06960	0.6297	0.8926	0.08366	0.3150	0.9952
	S_8_	f(x) = 1.258 + 1.142*exp(−exp(0.2264/1.142*(33.8-x) + 1))	33.8	0.08330	0.8533	0.2234	6.489	0.8652	0.2056	1.902	0.9363
	S_4_	f(x) = 0.982 + 2.285*exp(−exp(0.461/2.285*(12.12-x) + 1))	12.12	0.1696	0.8253	0.4219	23.14	0.8267	0.4099	7.562	0.9050
	PC 1	f(x) = −4.533 + 7.95*exp(−exp(0.3837/7.95*(8.262-x) + 1))	8.262	0.1412	0.9302	0.7606	75.20	0.9141	0.8216	30.38	0.9960
	lg(CFU/g)	f(x) = 2.83 + 7.652*exp(−exp(0.4287/7.652*(6.851-x) + 1))	6.851	0.1577	0.9988	0.1243	0.0772	—	—	—	—
Logistic	S_9_	f(x) = 0.5794 + 4.7/(1 + exp(0.06784*(38.81-x)))	38.81	0.06784	0.9550	0.3525	16.16	0.9434	0.3800	6.497	0.9869
	S_5_	f(x) = −1.084 + 0.839/(1 + exp(0.05719*(32.5-x)))	32.5	0.05719	0.9338	0.06832	0.6067	0.8933	0.0834	0.3133	0.9948
	S_8_	f(x) = 1.235 + 1.173/(1 + exp(0.2203*(41.44-x)))	41.44	0.2203	0.8585	0.2194	6.259	0.8674	0.2040	1.872	0.9524
	S_4_	f(x) = 1.006 + 2.236/(1 + exp(0.4148*(20.09-x)))	20.09	0.4148	0.8220	0.4259	23.58	0.8177	0.4205	7.956	0.8819
	PC 1	f(x) = −4.993 + 8.119/(1 + exp(0.07042*(34.88-x)))	34.88	0.07042	0.9333	0.7436	71.88	0.9170	0.8077	29.36	0.9950
	lg(CFU/g)	f(x) = 2.019 + 8.319/(1 + exp(0.07287*(28.68-x)))	28.68	0.07287	0.9996	0.07001	0.02451	—	—	—	—

**Table 4:** Results of fitting growth models for *P. aeruginosa* in fresh pork stored at 4 °C.

Method	Sensor	Simulation equation	λ (h)	μ_max_(h^−1^)	Training			Testing			r
					Rc^2^	RMSE_C_	SSE	Rp^2^	RMSE_P_	SSE	
Gompertz	S_9_	f(x) = 1.131 + 1.388*exp(−exp(0.06703/1.388*(102.3-x) + 1))	102.3	0.0247	0.8322	0.2764	12	0.8836	0.2784	3.797	0.9829
	S_4_	f(x) = 1.089 + 1.576*exp(−exp(0.07715/1.576*(113.6-x) + 1))	113.6	0.0284	0.7681	0.3787	22.52	0.8873	0.2720	3.626	0.9633
	PC 1	f(x) = −4.95 + 7.972*exp(−exp(0.1182/7.972*(−13.1-x) + 1))	−13.1	0.0435	0.7310	1.3770	297.8	0.8210	1.2482	76.35	0.9540
	lg(CFU/g)	f(x) = 3.383 + 4.455*exp(−exp(0.1169/4.455*(71.2-x) + 1))	71.2	0.0430	0.9861	0.2075	8.869	—	—		—
Logistic	S_9_	f(x) = 1.109 + 1.41/(1 + exp(0.06493*(132.8-x)))	132.8	0.06493	0.8450	0.2657	11.08	0.8991	0.2631	3.405	0.9889
	S_4_	f(x) = 1.089 + 1.557/(1 + exp(0.07956*(144.4-x)))	144.4	0.07956	0.7773	0.3712	21.26	0.9023	0.2550	3.186	0.9613
	PC 1	f(x) = −4.921 + 7.644/(1 + exp(0.02252*(81.05-x)))	81.05	0.02252	0.7357	1.365	292.6	0.8242	1.2367	74.95	0.9683
	lg(CFU/g)	f(x) = 3.307 + 4.306/(1 + exp(0.04258*(121.5-x)))	121.5	0.04258	0.9850	0.2152	9.543	—	—	—	—

## References

[b1] Gomes Neto N. J., Da Silva Luz I., Honório V. G., Da Conceição M. L. & de Souza E. L. *Pseudomonas Aeruginosa* cells adapted to *Rosmarinus Officinalis* L. essential oil and 1,8-Cineole acquire no direct and cross protection in a meat-Based broth. Food Res Int. 49, 143–146 (2012).

[b2] Yi S. M., Zhu J. L., Fu L. L. & Li J. R. Tea polyphenols inhibit *Pseudomonas Aeruginosa* through damage to the cell membrane. Int J Food Microbiol. 144, 111–117 (2010). 20884071 10.1016/j.ijfoodmicro.2010.09.005

[b3] Papadopoulou O. S., Panagou E. Z., Mohareb F. R. & Nychas G. E. Sensory and microbiological quality assessment of beef fillets using a portable electronic nose in tandem with support vector machine analysis. Food Res Int. 50, 241–249 (2013).

[b4] Gill C. O. . Evaluation of the hygienic performances of the processes for cleaning, dressing and cooling pig carcasses at eight packing plants. Int J Food Microbiol. 58, 65–72 (2000). 10898463 10.1016/s0168-1605(00)00294-4

[b5] Coates K. J., Beattie J. C., Morgan I. R. & Widders P. R. The contribution of carcass contamination and the boning process to microbial spoilage of aerobically stored pork. Food Microbiol. 12, 49–54 (1995).

[b6] Samelis J., Kakouri A. & Rementzis J. Selective effect of the product type and the packaging conditions on the species of lactic acid bacteria dominating the spoilage microbial association of cooked meats at 4 °C. Food Microbiol. 17, 329–340 (2000).

[b7] Egan A. F. & Roberts T. A. Microbiology of meat and meat products in Essays in Agricultural and Food Microbiology. (eds Norris J. R. & Pettipher. G. L.) 167–197 (1987).

[b8] Sun Y. . Growth simulation and discrimination of *Botrytis Cinerea*, *Rhizopus Stolonifer* and *Colletotrichum Acutatum* using hyperspectral reflectance imaging. PLoS One. 10(12), e0143400, doi: 10.1371/journal.pone.0143400 (2015). 26642054 PMC4671615

[b9] Ferrer J., Prats C., López D. & Vives-Rego J. Mathematical modelling methodologies in predictive food microbiology: A SWOT analysis. Int J Food Microbiol. 134, 2–8 (2009). 19217180 10.1016/j.ijfoodmicro.2009.01.016

[b10] Jiang Y. J. Construction of predictive growth model of *Pseudomonas* and *Escherichia coli* in chilled pork. Nanjing Agricultural University. pp: 8–35 (2008). (in Chinese with English abstract).

[b11] Zhang Y. M. Study of microbial growth kinetics model of *Pseudomonas* spp. and shelf life prediction for chilled beef. Shandong Agricultural University. pp: 9–37 (2010). (in Chinese with English abstract).

[b12] Wang X. . Modelling growth of *Pseudomonas aeruginosa* single cells with temperature shifts. J Food Safety. doi: 10.1111/jfs.12258 (2016).

[b13] Walter L., Knight G., Ng S. Y. & Buckow R. Kinetic models for pulsed electric field and thermal inactivation of *Escherichia Coli* and *Pseudomonas Fluorescens* in whole milk. Int Dairy J. 57, 7–14 (2016).

[b14] Li M. Y., Li Y. H., Huang X. Q., Zhao G. M. & Tian W. Evaluating growth models of *Pseudomonas* spp. in seasoned prepared chicken stored at different temperatures by the principal component analysis (PCA). Food Microbiol. 40, 41–47 (2014). 24549196 10.1016/j.fm.2013.11.014

[b15] Hong X. Z., Wang J. & Hai Z. Discrimination and prediction of multiple beef freshness indexes based on electronic nose. Sensor Actuat B-Chem. 161, 381–389 (2012).

[b16] Boothe D. D. H. & Arnold J. W. Electronic nose analysis of volatile compounds from poultry meat samples, fresh and after refrigerated storage. J Sci Food Agr. 82, 315–322 (2002).

[b17] Zaragozá P. . Evaluation of sea bream (*Sparus Aurata*) shelf life using an optoelectronic nose. Food Chem. 138, 1374–1380 (2013). 23411257 10.1016/j.foodchem.2012.10.114

[b18] Hu H. P., Pan Y. J., Liu Y., Sun X. H. & Zhao Y. Application of odor fingerprint for the detection of *Pseudomonas* spp. isolated from pork. Food Sci. 30, 327–332 (2009). (in Chinese with English abstract).

[b19] Bäck J. . Variable emissions of microbial volatile organic compounds (MVOCs) from root-associated fungi isolated from Scots pine. Atmos Environ. 44, 3651–3659 (2010).

[b20] Gutiérrez-Méndez N., Vallejo-Cordoba B., González-Córdova A. F., Nevárez-Moorillón G. V. & Rivera-Chavira B. Evaluation of aroma generation of *Lactococcus lactis* with an electronic nose and sensory analysis. J Dairy Sci. 91, 49–57 (2008). 18096924 10.3168/jds.2007-0193

[b21] McEntegart C. M., Penrose W. R., Strathmann S. & Stetter J. R. Detection and discrimination of coliform bacteria with gas sensor arrays. Sensor Actuat B-Chem. 70, 170–176 (2000).

[b22] Lippolis V. . Rapid prediction of ochratoxin A-producing strains of *Penicillium* on dry-cured meat by MOS-based electronic nose. Int J Food Microbiol. 218, 71–77 (2016). 26619315 10.1016/j.ijfoodmicro.2015.11.011

[b23] Wang H. X. . Early detection of *Zygosaccharomyces Rouxii*—spawned spoilage in apple juice by electronic nose combined with chemometrics. Int J Food Microbiol. 217, 68–78 (2016). 26490651 10.1016/j.ijfoodmicro.2015.10.010

[b24] Aguirre J. S. & Koutsoumanis K. P. Towards lag phase of microbial populations at growth-limiting conditions: the role of the variability in the growth limits of individual cells. INT J Food Microbiol. 224, 1–6 (2016). 26900994 10.1016/j.ijfoodmicro.2016.01.021

[b25] Qiu J. Modeling *Pseudomonas* spp. growth in the modified atmosphere packaged chilled pork and package optimization. University of Shanghai for Science and Technology. pp: 14–42 (2012). (in Chinese with English abstract)

[b26] Pan L. Q., Zhang W., Zhu N., Mao S. B. & Tu K. Early detection and classification of pathogenic fungal disease in post-harvest strawberry fruit by electronic nose and gas chromatography–mass spectrometry. Food Res Int. 62, 162–168 (2014).

[b27] Nguimkeu P. A simple selection test between the Gompertz and Logistic growth models. Technol Forecast Soc. 88, 98–105 (2014).

[b28] Chatterjee T., Chatterjee B. K., Majumdar D. & Chakrabarti P. Antibacterial effect of silver nanoparticles and the modeling of bacterial growth kinetics using a modified Gompertz model. BBA-Gen Subjects. 1850, 299–306 (2015). 10.1016/j.bbagen.2014.10.022 25450183

[b29] Peleg M., Corradini M. G. & Normand M. D. The logistic (Verhulst) model for sigmoid microbial growth curves revisited. Food Res Int. 40, 808–818 (2007).

[b30] GB 4789.3-2010: Food microbiological examination: enumeration of coliforms. China (2010).

[b31] Melucci D. . Rapid direct analysis to discriminate geographic origin of extra virgin olive oils by flash gas chromatography electronic nose and chemometrics. Food Chem. 204, 263–273 (2016). 26988501 10.1016/j.foodchem.2016.02.131

[b32] Neves P. R., McCulloch J. A., Mamizuka E. M. & Lincopan N. Pseudomonas Aeruginosa In Encyclopedia of Food Microbiology (Second Edition). (ed. Batt C. A.) 253–260, 10.1016/B978-0-12-384730-0.00283-4 (2014).

[b33] Lentini G. . Rapid detection of *Pseudomonas Aeruginosa* by phage-capture system coupled with micro-Raman spectroscopy. Vib Spectrosc. 86, 1–7 (2016).

[b34] N. Magana P. E. Volatiles as an indicator of fungal activity and differentiation between species, and the potential use of electronic nose technology for early detection of grain spoilage. J Stored Prod Res. 36, 319–340 (2000). 10880811 10.1016/s0022-474x(99)00057-0

[b35] Wang D. F. Research on the odor fingerprints of five main harmful microorganisms of chilled pork. Shanghai Ocean University. pp: 3–36 (2010). (in Chinese with English abstract).

[b36] Federico B. . Efficacy of lactoferricin B in controlling ready-to-eat vegetable spoilage caused by *Pseudomonas* spp. Int J Food Microbiol. 215, 179–186 (2015). 26453993 10.1016/j.ijfoodmicro.2015.09.017

[b37] Caldera L. . Identification, enzymatic spoilage characterization and proteolytic activity quantification of *Pseudomonas* spp. isolated from different foods. Food Microbiol. 54, 142–153 (2016).

[b38] Zaragozá P. . Evaluation of sea bream (*Sparus aurata*) shelf life using an optoelectronic nose. Food Chem. 138, 1374–1380 (2013). 23411257 10.1016/j.foodchem.2012.10.114

[b39] Salinas Y. . A novel colorimetric sensor array for monitoring fresh pork sausages spoilage. Food Control. 35, 166–176 (2014).

[b40] Peng Y. Study on the putrefaction potential of putrescence microorganism in chilled pork. China Agricultural University. pp: 7–35 (2005). (in Chinese with English abstract).

[b41] Gill C. O. & Newton K. G. The development of aerobic spoilage flora on meat stored at chill temperatures. J. Appl. Bacteriol. 43, 189–195 (1977). 22531 10.1111/j.1365-2672.1977.tb00742.x

[b42] Gill C. O. & Newton K. G. The ecology of bacterial spoilage of fresh meat at chill temperatures. Meat Sci. 2, 207–217 (1978). 22055052 10.1016/0309-1740(78)90006-2

[b43] Zaragozá P. . Monitorization of Atlantic salmon (*Salmo Salar*) spoilage using an optoelectronic nose. Sensor Actuat B-Chem. 195, 478–485 (2014).

