# Supplementary material for: Predicting the growth situation of Pseudomonas aeruginosa on agar plates and meat stuffs using gas sensors
Source: Sci Rep. 2016 Dec 12;6:38721. doi: 10.1038/srep38721 (PMC5150633; doi:10.1038/srep38721)
Supplement: Supplementary Information [file srep38721-s1.pdf]

Supplementary information files

Predicting the growth situation of *Pseudomonas aeruginosa* on agar plates and meat stuff using gas sensors

Xinzhe Gu[1], Ye Sun[1], Kang Tu[1], Qingli Dong[2] & Leiqing Pan[1]*

[1]College of Food Science and Technology, Nanjing Agricultural University, No.1, Weigang Road, Nanjing, Jiangsu, 210095, PR China

[2]School of Medical Instrument and Food Engineering, University of Shanghai for Science and Technology, 516 Jun Gong Rd., Shanghai 200093, PR China

***Corresponding author:**

Leiqing Pan, Ph.D. Associate professor

College of Food Science and Technology

Nanjing Agricultural University

NO.1 Weigang Road, Nanjing, 210095, P.R. China

Tel.: +86-025-84399016

Email: pan_leiqing@njau.edu.cn

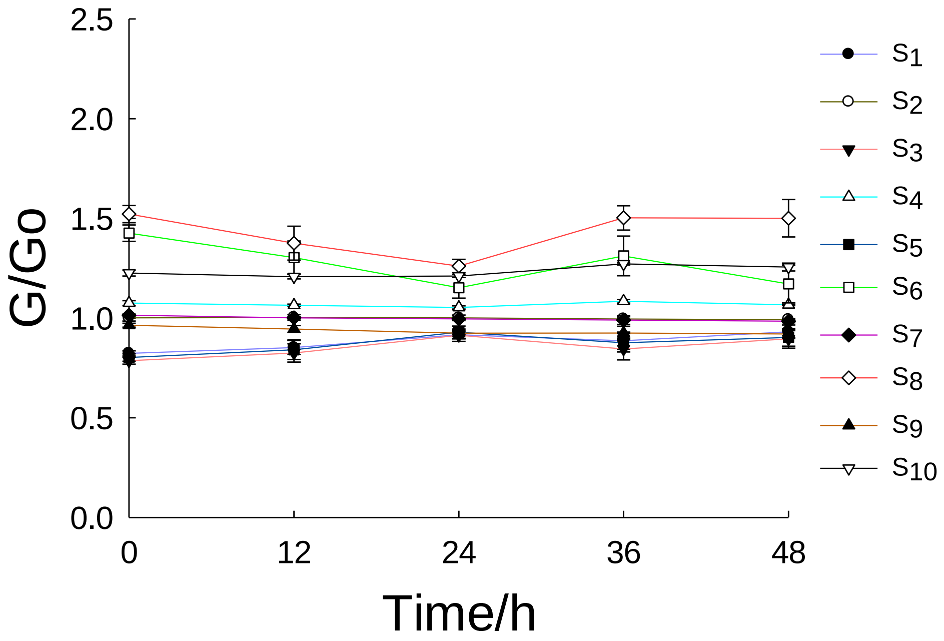

Supplementary Fig. A1. Average response values of 10 sensors of the control group for agar plates during the inoculation of 48 h

Refer to **Supplementary Fig. A1** at the point of '*Results/Growth simulation of P. aeruginosa on agar plate by gas sensors/ Gas sensors response.*

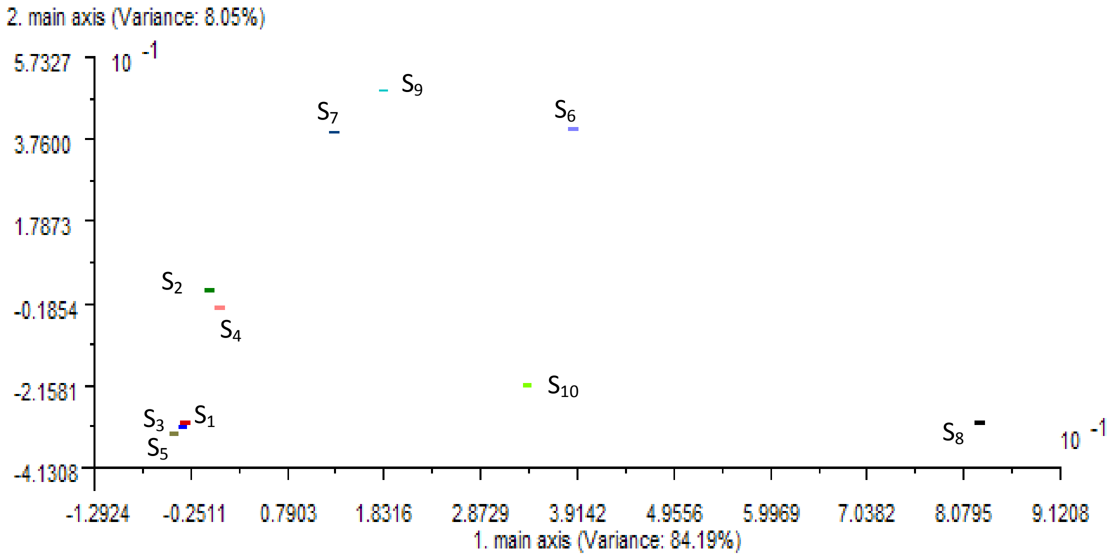

Supplementary Fig. A2. Loading analysis of *P. aeruginosa* on the agar plates

Refer to **Supplementary Fig. A2** at the point of '*Results/Growth simulation of P. aeruginosa on agar plate by gas sensors/ Sensors selection*'.

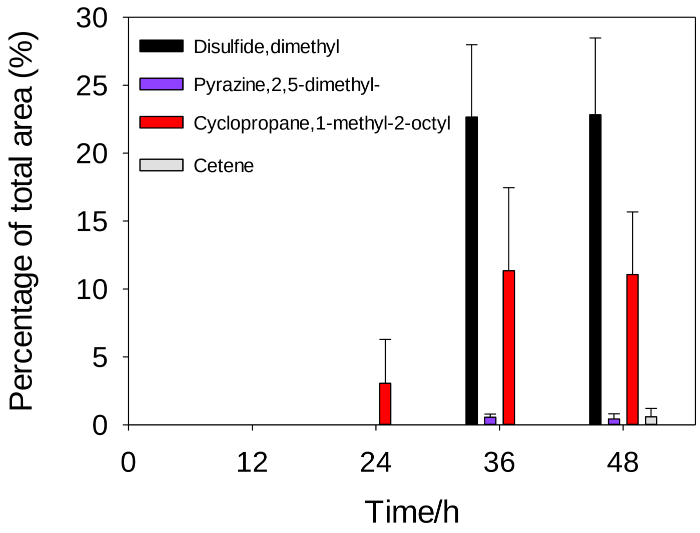

Supplementary Fig. A3. Some volatile compounds of agar plate samples inoculated with P. aeruginosa during 48 h of incubation.

Refer to **Supplementary Fig. A3** at 'HS-SPME/GC–MS analysis'.

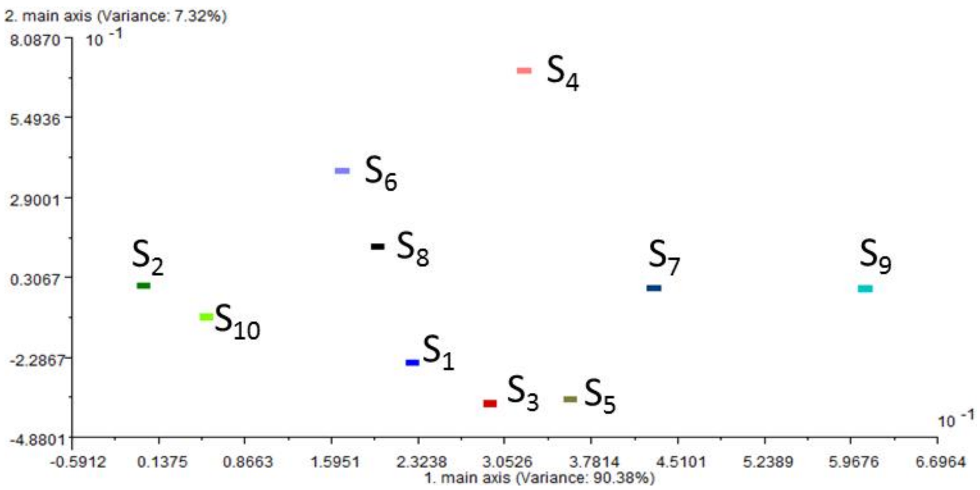

Supplementary Fig. A4. Loading analysis of *P. aeruginosa* in fresh pork at 20 ℃

Refer to **Supplementary Fig. A4** at the point of '*Results/ Growth simulation of P. aeruginosa inoculated in fresh pork by gas sensors/ Sensor selection for growth simulation of P. aeruginosa in fresh pork stored at 20 ℃*'.

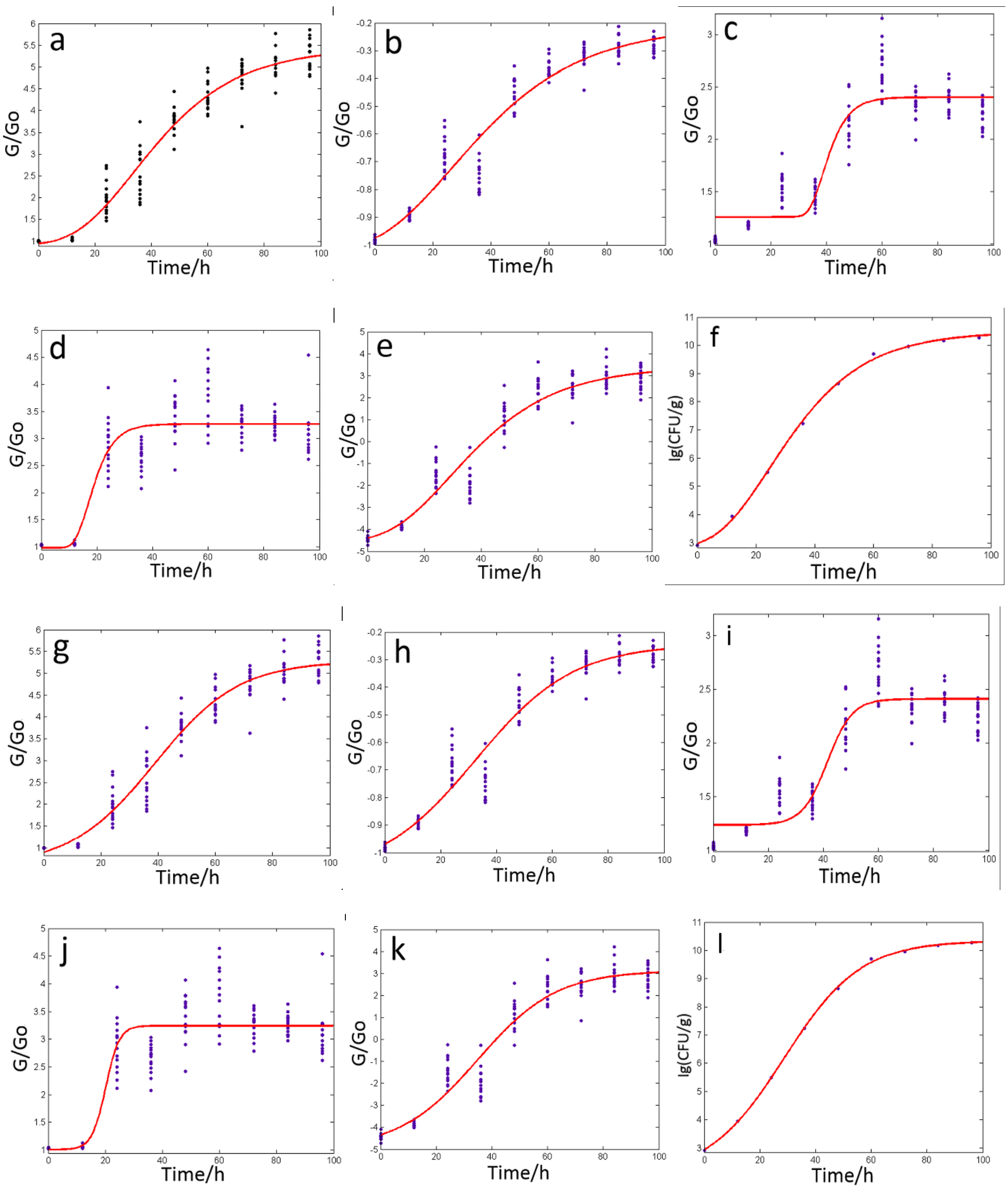

Supplementary Fig. A5. Fitting the growth curve of for *P. aeruginosa* in fresh pork stored at 20℃ (a: S_9, Gompertz; b: S_5, Gompertz; c: S_8, Gompertz; d: S_4, Gompertz; e: PC 1, Gompertz; f: lg(CFU/g), Gompertz; g: S_9, Logistic; h: S_5, Logistic; i: S_8, Logistic; j: S_4, Logistic; k: PC 1, Logistic; l: lg(CFU/g), Logistic)

Refer to **Supplementary Fig. A5** at the point of '*Results/ Growth simulation of P. aeruginosa inoculated in fresh pork by gas sensors/ Growth simulation of P. aeruginosa in fresh pork stored at 20 ℃*'.

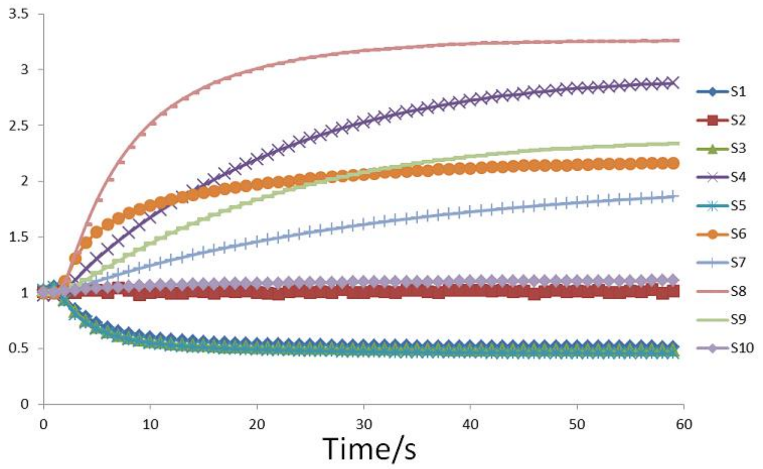

Supplementary Fig. A6. Response values of 10 sensors of a meat sample inoculated with *P. aeruginosa* at 168 h stored at 4 ℃

Refer to **Supplementary Fig. A6** at the point of *'Results/ Growth simulation of P. aeruginosa inoculated in fresh pork by gas sensors/ Growth simulation of P. aeruginosa in fresh pork stored at 4 ℃'*.

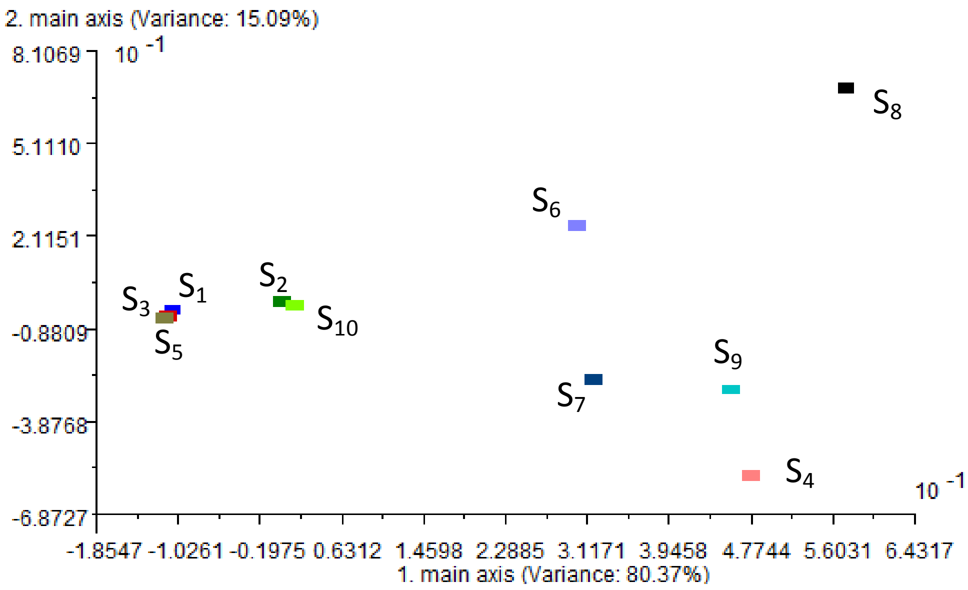

Supplementary Fig. A7. Loading analysis of *P. aeruginosa* in fresh pork at 4 ℃

Refer to **Supplementary Fig. A7** at the point of *'Results/ Growth simulation of P. aeruginosa inoculated in fresh pork by gas sensors/ Growth simulation of P. aeruginosa in fresh pork stored at 4 ℃'*.

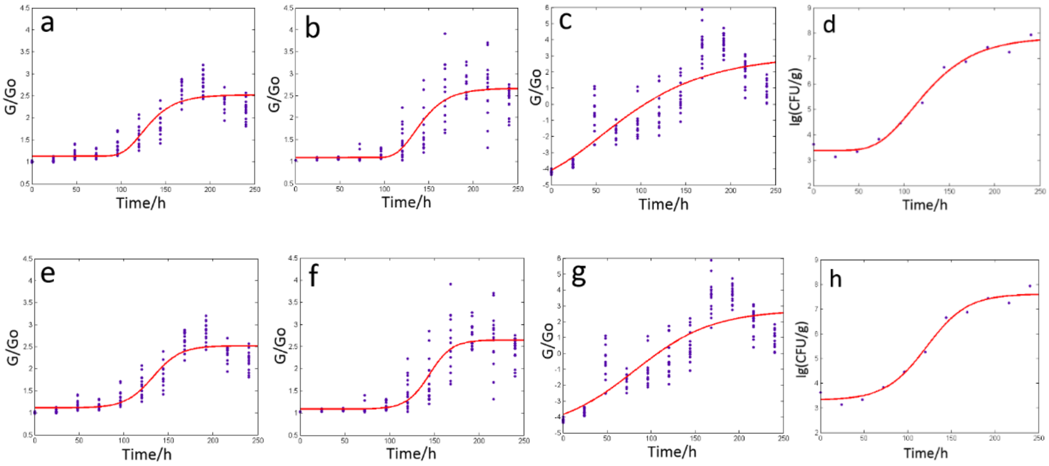

Supplementary Fig. A8. Fitting the growth curve of for *P. aeruginosa* in fresh pork stored at 4℃

(a: S_9, Gompertz; b: S_4, Gompertz; c: PC 1, Gompertz; d: lg(CFU/g), Gompertz; e: S_9, Logistic; f: S_4, Logistic; g: PC 1, Logistic; h: lg(CFU/g), Logistic)

Refer to **Supplementary Fig. A8** at the point of '*Results/ Growth simulation of P. aeruginosa inoculated in fresh pork by gas sensors/ Growth simulation of P. aeruginosa in fresh pork stored at 4℃*.

Supplementary Table B1. Variance analysis of *P. aeruginosa* on agar plates

	S_1	S_2	S_3	S_4	S_5	S_6	S_7	S_8	S_9	S_{10}
0 h	0.798± 0.019d	1.001±0. 018ab	0.744±0. 025d	1.056±0 .007b	0.746±0 .029e	1.489±0 .065b	1.007±0 .006d	1.593±0 .08d	0.974±0 .023d	1.194± 0.009c
12 h	0.936± 0.032a	1.004±0. 016a	0.941±0. 048a	1.058±0 .006b	0.958±0 .05a	1.035±0 .085c	1.004±0 .003d	1.521±0 .068d	0.936±0 .013d	1.340± 0.022b
24 h	0.826± 0.030c	1.008±0. 014a	0.850±0. 022bc	1.056±0 .007b	0.850±0 .022c	1.511±0 .083b	1.303±0 .09a	2.339±0 .097c	1.354±0 .111a	1.701± 0.053a
36 h	0.833± 0.069c	0.997±0. 011ab	0.822±0. 055c	1.061±0 .006b	0.811±0 .061d	1.710±0 .114a	1.190±0 .1b	2.558±0 .128b	1.193±0 .112b	1.703± 0.056a
48 h	0.906± 0.034b	0.992±0. 017b	0.875± 0.028b	1.079±0 .026a	0.879±0 .029b	1.649±0 .159a	1.064±0 .049c	2.708±0 .239a	1.060±0 .044c	1.737± 0.086a

Values (mean ± standard deviation) are statistically different in the same column with different letter.

Refer to **Supplementary Table B1** at the point of '*Results/Growth simulation of P. aeruginosa on agar plate by gas sensors/ Sensors selection*'.

Supplementary Table B2. Pearson correlation analysis of *P. aeruginosa* on agar plates.

	S_1	S_2	S_3	S_4	S_5	S_6	S_7	S_8	S_9	S_{10}	lgCFU
S_1	1										
S_2	-0.043	1									
S_3	0.909**	0.025	1								
S_4	0.243*	-0.179	0.096	1							
S_5	0.913**	0.035	0.995**	0.081	1						
S_6	-0.55**	-0.22*	-0.624**	0.298**	-0.676**	1					
S_7	-0.569**	0.077	-0.237*	-0.216*	-0.281**	0.411**	1				
S_8	-0.165	-0.235*	-0.146	0.389**	-0.213*	0.842**	0.486**	1			
S_9	-0.576**	0.066	-0.272*	-0.177	-0.319**	0.482**	0.991**	0.547**	1		
S_{10}	-0.042	-0.159	0.081	0.322**	0.009	0.669**	0.597**	0.946**	0.639**	1	
lgCFU	0.151	-0.159	0.246*	0.333**	0.177	0.525**	0.470**	0.885**	0.499**	0.942**	1

*: significant difference (P<0.05); **: extremely significant difference (P<0.01)

Refer to **Supplementary Table B2** at the point of '*Results/Growth simulation of P. aeruginosa on agar plate by gas sensors/ Sensors selection*'.

Supplementary Table B3. Volatile compounds identified by HS-SPME/GC–MS analysis of the control group (agar plate samples) during the incubation of 48 h.

No	r_t[a]	Volatile Compound	0 h	12 h	24 h	36 h	48 h
1	7.735	Oxime-,methoxy-phenyl-	+[b]	-[c]	-	-	-
2	9.269	Benzaldehyde	+	+	-	+	-
3	10.766	Octanal	+	-	-	-	-
4	14.144	Nonanal	+	-	-	+	-
5	17.268	Decanal	+	+	+	-	+
6	22.23	Undecanal	+	-	-	-	-
7	13.988	Undecane	+	-	-	-	+
8	19.965	Tridecane	-	-	-	+	+
9	26.172	Pentadencane	+	+	-	+	+
10	22.975	Hexagecane	+	-	-	-	+

[a] r_t: retention time; [b] +: detected; [c] -: not detected.

Refer to **Supplementary Table B3** at the point of '*Results/ Growth simulation of P. aeruginosa on agar plate by gas sensors/ HS-SPME/GC–MS analysis*'.

Supplementary Table B4. Variance analysis of *P. aeruginosa* in fresh pork at 20 ℃

	S_1	S_2	S_3	S_4	S_5	S_6	S_7	S_8	S_9	S_{10}
0 h	0.982±0.008a	1.007±0.013ab	0.993±0.01a	1.029±0.007f	0.983±0.01a	1.014±0.013h	1.005±0.006h	1.039±0.019h	0.996±0.009i	1.061±0.005f
12 h	0.892±0.027b	1±0.012b	0.892±0.026b	1.059±0.023f	0.886±0.031b	1.126±0.021g	1.05±0.077h	1.179±0.024g	1.065±0.108h	1.075±0.003ef
24 h	0.707±0.054c	1.005±0.015ab	0.71±0.059c	2.845±0.433d	0.659±0.067c	1.827±0.18e	1.69±0.293g	1.566±0.124e	2.067±0.394g	1.088±0.006e
36 h	0.799±0.038d	1.005±0.016ab	0.804±0.039d	2.603±0.261e	0.75±0.058d	1.636±0.13f	1.944±0.4f	1.449±0.095f	2.501±0.533f	1.024±0.005g
48 h	0.587±0.07e	1.008±0.009ab	0.57±0.083e	3.43±0.396b	0.465±0.066e	2.671±0.263a	2.961±0.233e	2.159±0.182d	3.818±0.273e	1.14±0.013d
60 h	0.483±0.035f	1.013±0.011a	0.44±0.037f	3.686±0.534a	0.364±0.033f	2.53±0.307b	3.283±0.242d	2.669±0.232a	4.305±0.37d	1.212±0.045c
72 h	0.425±0.042g	1.014±0.012a	0.375±0.046g	3.242±0.241bc	0.325±0.041g	2.178±0.171c	3.582±0.244c	2.297±0.131c	4.72±0.352c	1.37±0.063b
84 h	0.384±0.032h	1.014±0.016a	0.326±0.035h	3.326±0.221bc	0.287±0.035h	2.158±0.132c	3.762±0.215b	2.407±0.117b	5.03±0.321b	1.482±0.054a
96 h	0.365±0.025h	1.012±0.011a	0.312±0.026h	3.142±0.416c	0.283±0.029h	1.996±0.113d	4.038±0.464a	2.245±0.13c	5.255±0.33a	1.463±0.048a

Values (mean ± standard deviation) are statistically different in the same column with different letter.

Refer to **Supplementary Table B4** at the point of '*Results/ Growth simulation of P. aeruginosa inoculated in fresh pork by gas sensors/ Sensor selection for growth simulation of P. aeruginosa in fresh pork stored at 20 ℃*'.

Supplementary Table B5. Pearson correlation analysis of *P. aeruginosa* in fresh pork at 20 ℃

	S_1	S_2	S_3	S_4	S_5	S_6	S_7	S_8	S_9	S_{10}	lg(CFU)
S_1	1										
S_2	-0.284**	1									
S_3	0.999**	-0.287**	1								
S_4	-0.815**	0.276**	-0.801**	1							
S_5	0.995**	-0.290**	0.993**	-0.846**	1						
S_6	-0.760**	0.252**	-0.746**	0.931**	-0.809**	1					
S_7	-0.964**	0.282**	-0.964**	0.816**	-0.965**	0.764**	1				
S_8	-0.916**	0.303**	-0.916**	0.892**	-0.937**	0.897**	0.905**	1			
S_9	-0.970**	0.294**	-0.969**	0.826**	-0.973**	0.782**	0.994**	0.917**	1		
S_{10}	-0.866**	0.296**	-0.877**	0.533**	-0.831**	0.444**	0.848**	0.717**	0.849**	1	
lg(CFU)	-0.943**	0.282**	-0.941**	0.856**	-0.955**	0.806**	0.949**	0.916**	0.962**	0.768**	1

*: significant difference ($P<0.05$); **: extremely significant difference ($P<0.01$)

Refer to **Supplementary Table B5** at the point of '*Results/ Growth simulation of P. aeruginosa inoculated in fresh pork by gas sensors/ Sensor selection for growth simulation of P. aeruginosa in fresh pork stored at 20 ℃*'.

Supplementary Table B6. Variance analysis of *P. aeruginosa* in fresh pork at 4 ℃

	S_1	S_2	S_3	S_4	S_5	S_6	S_7	S_8	S_9	S_{10}
0h	0.978±0.007a	1.002±0.009ab	0.989±0.008a	1.029±0.003e	0.978±0.01a	1.024±0.013f	1.005±0.006h	1.052±0.017f	0.996±0.011i	1.062±0.005e
24h	0.921±0.021b	1.001±0.014ab	0.926±0.024b	1.065±0.042e	0.919±0.022b	1.091±0.032f	1.039±0.042h	1.132±0.038f	1.052±0.062hi	1.073±0.005d
48h	0.635±0.074e	1.004±0.011ab	0.587±0.083e	1.066±0.02e	0.563±0.092e	1.86±0.309bc	1.056±0.034h	2.305±0.493c	1.157±0.106h	1.058±0.014e
72h	0.727±0.035c	0.998±0.016b	0.678±0.043c	1.091±0.102e	0.646±0.05c	1.509±0.1e	1.075±0.056h	1.963±0.253e	1.159±0.069h	1.056±0.009e
96h	0.69±0.057d	1.003±0.015ab	0.638±0.06d	1.155±0.098e	0.596±0.063d	1.605±0.222de	1.2±0.103g	2.321±0.489c	1.366±0.167g	1.052±0.01ef
120h	0.646±0.053e	1.005±0.012ab	0.6±0.058e	1.418±0.319d	0.561±0.059e	1.63±0.26de	1.395±0.183f	2.254±0.518cd	1.598±0.228f	1.041±0.011g
144h	0.588±0.046f	1.001±0.014ab	0.542±0.052f	1.691±0.443c	0.503±0.055f	1.848±0.278bc	1.578±0.251e	2.804±0.667b	1.853±0.235e	1.044±0.013fg
168h	0.481±0.027h	1.002±0.009ab	0.451±0.03h	2.52±0.579b	0.424±0.031h	2.222±0.293a	2.174±0.211b	3.459±0.573a	2.675±0.221b	1.113±0.032b
192h	0.465±0.022h	1.007±0.015ab	0.439±0.025h	2.859±0.35a	0.415±0.026h	2.246±0.225a	2.321±0.21a	3.351±0.559a	2.888±0.224a	1.131±0.027a
216h	0.535±0.027g	1.009±0.012a	0.505±0.029g	2.654±0.564ab	0.469±0.031g	1.903±0.163b	1.988±0.16c	2.286±0.249cd	2.421±0.188c	1.091±0.014c
240h	0.571±0.04h	1.003±0.011ab	0.542±0.043f	2.461±0.298b	0.505±0.046f	1.734±0.113cd	1.858±0.224d	2.004±0.17de	2.241±0.264d	1.075±0.015d

Values (mean ± standard deviation) are statistically different in the same column with different letter.

Refer to **Supplementary Table B6** at the point of '*Results/ Growth simulation of P. aeruginosa inoculated in fresh pork by gas sensors/ Growth simulation of P. aeruginosa in fresh pork stored at 4 ℃*'.

Supplementary Table B7. Pearson correlation analysis of *P. aeruginosa* in fresh pork at 4 ℃

	S_1	S_2	S_3	S_4	S_5	S_6	S_7	S_8	S_9	S_{10}	lg(CFU)
S_1	1										
S_2	-0.109	1									
S_3	0.996**	-0.101	1								
S_4	-0.712**	0.144*	-0.663**	1							
S_5	0.991**	-0.099	0.999**	-0.648**	1						
S_6	-0.920**	0.096	-0.916**	0.706**	-0.908**	1					
S_7	-0.814**	0.138*	-0.762**	0.849**	-0.747**	0.719**	1				
S_8	-0.858**	0.066	-0.861**	0.586**	-0.855**	0.958**	0.661**	1			
S_9	-0.850**	0.139*	-0.802**	0.866**	-0.787**	0.770**	0.993**	0.712**	1		
S_{10}	-0.440**	0.089	-0.380**	0.742**	-0.349**	0.543**	0.662**	0.477**	0.675**	1	
lg(CFU)	-0.788**	0.110	-0.751**	0.848**	-0.748**	0.647**	0.879**	0.578**	0.896**	0.462**	1

*: significant difference (P<0.05); **: extremely significant difference (P<0.01)

Refer to **Supplementary Table B7** at the point of '*Results/ Growth simulation of P. aeruginosa inoculated in fresh pork by gas sensors/ Growth simulation of P. aeruginosa in fresh pork stored at 4 ℃*'.

Supplementary Table B8. Response material and sensitivity of 10 metal oxide sensors

Sensor	Response material	Sensitivity
S_1	Sensitive to aromatic compounds	10ppm
S_2	Sensitive to nitrogen oxides	NO_2,1ppm
S_3	Sensitive to ammonia and aromatic compounds	Benzene,10ppm
S_4	Sensitive to hydrogen	H_2,100ppb
S_5	Sensitive to alkenes and aromatic compounds	Propane,1ppm
S_6	Sensitive to methane broad range	CH_3,100ppm
S_7	Sensitive to sulphur compounds	H_2S,1ppm
S_8	Sensitive to alcohols and partially aromatic compounds	CO,100ppm
S_9	Sensitive to aromatics compounds and sulphur organic compounds	H_2S,1ppm
S_{10}	Sensitive to alkane	CH_3,100ppm

Refer to **Supplementary Table B8** at the point of '*Materials and methods/ Electronic nose trials*'.
